# Non-suppurative Destructive Cholangitis After Avacopan Therapy in Myeloperoxidase-Antineutrophil Cytoplasmic Antibody (MPO-ANCA)-Associated Glomerulonephritis: A Case Report and Review of the Literature

**DOI:** 10.7759/cureus.114585

**Published:** 2026-08-16

**Authors:** Kensuke Terakawa, Yukihiro Wada, Yuki Toyoda, Keiya Nakamura, Rina Kamiya, Ryota Uchitsubo, Shun Sakurabayashi, Tomomi Motohashi, Sayumi Kawamura,, Hiroshi Tominaga, Kazuhiro Takeuchi, Yutaro Saito, Masataka Tochimoto, Hisashi Hidaka, Makoto Saegusa, Yasuo Takeuchi

**Affiliations:** 1 Department of Nephrology, Kitasato University School of Medicine, Sagamihara, JPN; 2 Department of Gastroenterology, Kitasato University School of Medicine, Sagamihara, JPN; 3 Department of Pathology, Kitasato University School of Medicine, Sagamihara, JPN

**Keywords:** avacopan, cholangitis, microscopic polyangiitis, mpo-anca, non-suppurative destructive cholangitis

## Abstract

Avacopan, a selective complement C5a receptor antagonist, is utilized to manage microscopic polyangiitis (MPA). Recently, attention has grown regarding severe liver injury, particularly vanishing bile duct syndrome (VBDS), as a potential adverse event during avacopan therapy. However, its clinicopathological features and underlying mechanisms remain poorly understood. Herein, we report a case of non-suppurative destructive cholangitis (NSDC), considered a pre-conditional state of VBDS, after avacopan therapy for MPA.

A 55-year-old obese female with myeloperoxidase-antineutrophil cytoplasmic antibody (MPO-ANCA)-associated crescentic glomerulonephritis achieved remission via steroid pulse therapy, oral prednisolone (PSL), and rituximab. Seven weeks before admission, avacopan and ursodeoxycholic acid (UDCA) were initiated. Despite stable renal function, MPO-ANCA seroconversion to negative, and successful PSL tapering, she presented with acute liver injury. Laboratory tests revealed marked elevations in transaminases and biliary enzymes (aspartate aminotransferase (AST): 313 U/L, alanine aminotransferase (ALT): 488 U/L, gamma-glutamyl transferase (γ-GTP): 364 U/L) with normal direct bilirubin (D-bil). Avacopan was discontinued, and PSL was increased. A liver biopsy showed lymphocytic (non-suppurative) destructive cholangitis with a florid duct lesion and frequent spotty necrosis in lobuli, without central necrosis. Immunohistochemical staining revealed focal decreased CK19 immunointensity in the bile ducts and predominant infiltration of CD4-positive T cells and CD68-positive macrophages around interlobular bile ducts. Although D-bil transiently peaked at 4.0 mg/dL on day 7, intensive treatment with high-dose UDCA and intravenous glycyrrhizin restored D-bil to the normal range by day 34, with significant transaminase improvement.

Pathological findings revealed biliary epithelial damage with predominant T-cell and macrophage infiltration, distinct from typical drug-induced liver injury. The onset during PSL tapering and responsiveness to temporary PSL intensification strongly support a cell-mediated immune mechanism driving VBDS. To the best of our knowledge, this is the first report describing a comprehensive immunohistochemical evaluation of the periportal microenvironment in avacopan-induced biliary injury. This case highlights that severe cholangitis can occur regardless of baseline risk profiles or disease activity, underscoring the need for vigilant, long-term monitoring of liver enzymes and bilirubin levels during avacopan therapy.

## Introduction

Anti-neutrophil cytoplasmic antibody (ANCA)-associated vasculitis (AAV) is a systemic necrotizing small-vessel vasculitis [1,2]. Microscopic polyangiitis (MPA) is the predominant subtype, typically presenting in Japanese patients as myeloperoxidase (MPO)-ANCA-associated crescentic glomerulonephritis (ANCA-associated GN), a major cause of rapidly progressive glomerulonephritis (RPGN) leading to severe renal damage [2,3]. Although high-dose glucocorticoids (GCs) with cytotoxic agents or rituximab (Rit) are standard induction therapies, prolonged GC use causes substantial adverse effects, organ toxicity, and infection-related mortality.

Avacopan, a selective antagonist of complement C5a receptor 1 (C5aR1), has emerged as an effective alternative to GCs for induction therapy in AAV [4]. C5aR1 is expressed on neutrophils, monocytes, and vascular endothelial cells [5]. In AAV pathogenesis, the C5a-C5aR1 interaction acts synergistically with ANCAs to activate neutrophils, inducing neutrophil extracellular trap (NET) formation and alternative complement pathway activation, thereby creating a vicious inflammatory cycle [6]. By disrupting this cascade, avacopan achieves effective disease control [7]. Previous randomized controlled studies, including the representative ADVOCATE trial, demonstrated that avacopan combined with cyclophosphamide or Rit achieved superior remission rates and significantly lower GC-associated toxicity compared with standard GC regimens [7,8]. Consequently, clinical guidelines recommend avacopan to facilitate early GC reduction or withdrawal [9].

However, several cases of avacopan-induced liver injury have been reported recently [10]. While retrospective studies outside Japan showed that the incidence of transient liver injury during avacopan therapy was not significant [11-13], single-center studies in Japan have revealed a notably higher incidence of liver injury ranging from 16.7% to 38.1% [14-16]. Furthermore, a striking cluster of drug-induced liver injury (DILI) cases has emerged within a short period in Japan, including severe presentations of vanishing bile duct syndrome (VBDS) [14-24]. These observations raise two critical clinical questions: which patients are at high risk for developing this severe hepatotoxicity, and what is its most plausible mechanism?

Herein, we report a case of severe, non-suppurative destructive cholangitis (NSDC) induced by avacopan in a patient with MPA. To the best of our knowledge, this is the first published case report describing a comprehensive immunohistochemical evaluation of the inflamed periportal microenvironment in avacopan-induced biliary injury. In this article, we comprehensively review seven previously reported　cases of biopsy-proven avacopan-induced cholangitis alongside our present case (a total of eight cases). By analyzing their detailed clinicopathological features, we aim to elucidate the clinical heterogeneity and underlying cellular mechanisms of this critical adverse effect, thereby providing pivotal insights to prevent further severe hepatotoxicity associated with avacopan therapy.

## Case presentation

**Figure 1:**
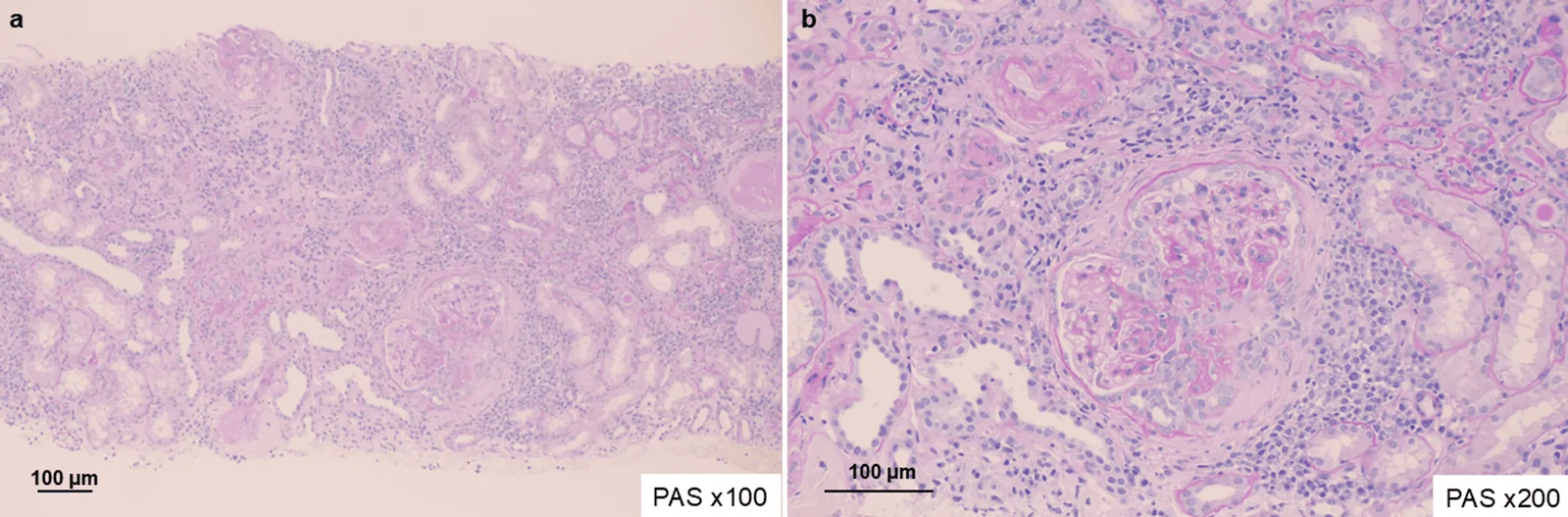
Light microscopy findings of the renal biopsy specimens. (a) Overview of the renal biopsy sample shows a glomerulus with crescentic formation and global/segmental sclerotic changes, accompanied by diffuse mononuclear cell infiltration in the interstitium (periodic acid-Schiff (PAS) stain; original magnification ×100). (b) A glomerulus exhibits crescentic glomerulonephritis characterized by a prominent cellular to fibrocellular crescent with rupture of the glomerular capillary loops (PAS stain; original magnification ×200).

**Figure 2:**
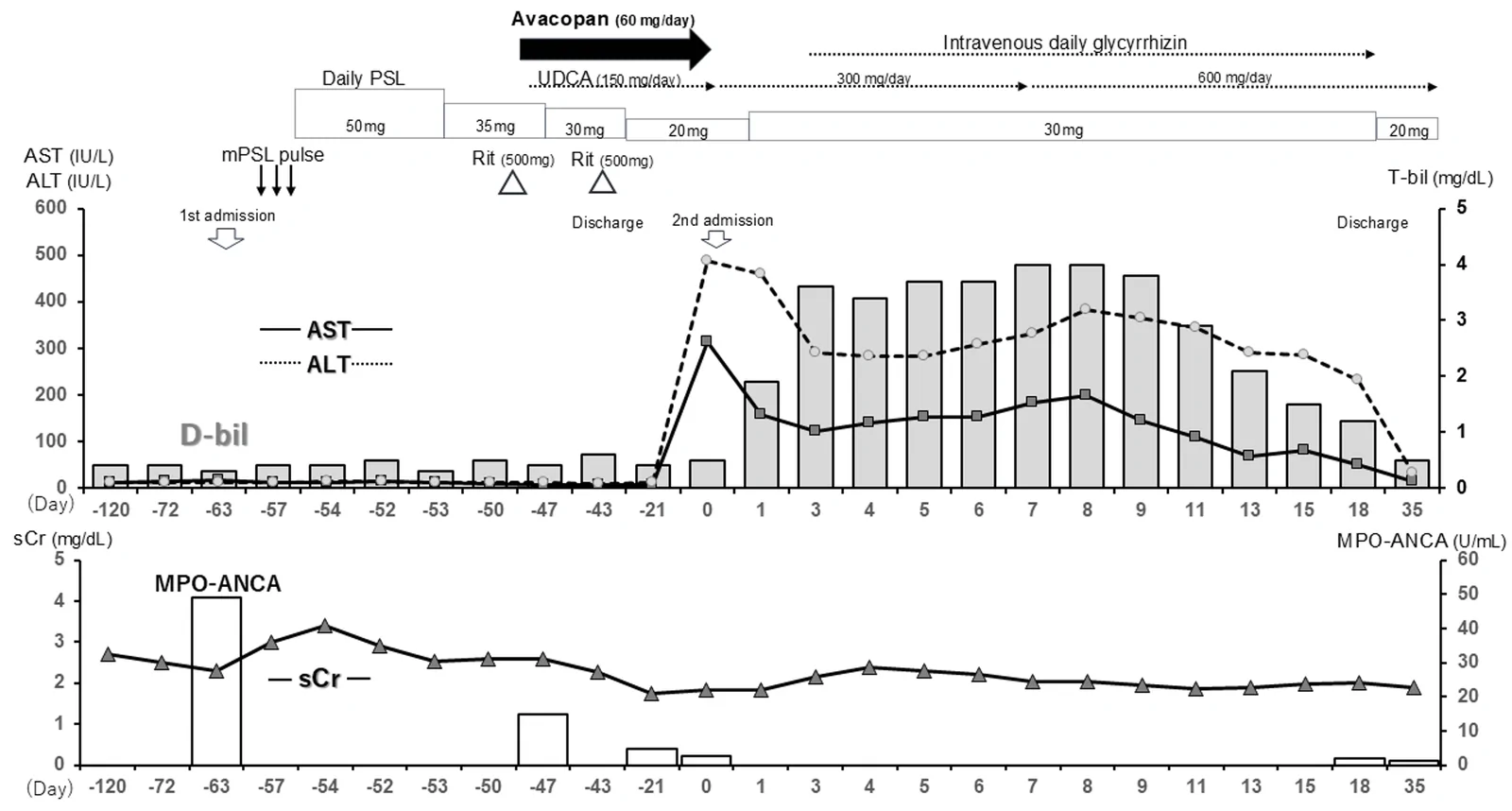
Clinical course and treatment of the patient before and after avacopan therapy. ALT, alanine aminotransferase; AST, aspartate aminotransferase; mPSL, methylprednisolone; MPO-ANCA, myeloperoxidase-antineutrophil cytoplasmic antibody; PSL, prednisolone; Rit, rituximab; sCr, serum creatinine; T-bil, total bilirubin; U, unit; UDCA, ursodeoxycholic acid The image was generated using Microsoft Excel and Microsoft PowerPoint (Microsoft Corporation, Redmond, WA).

A 55-year-old woman was urgently readmitted to our hospital due to severe liver injury. Four months before this readmission, she had been noted to have renal dysfunction with a serum creatinine (sCr) level of 2.7 mg/dL, accompanied by mild-to-moderate proteinuria and hematuria, during a routine health check-up. Although she had no prior history of renal disease on annual examinations, her medical history was remarkable except for resolved cholelithiasis and anxiety disorder, for which she was receiving an anxiolytic medication. Two months before readmission, she was referred to our hospital for further examination and treatment of her renal dysfunction. Upon her first admission, her sCr level was 2.3 mg/dL, accompanied by nephrotic-range proteinuria (3.72 g/gCr) and microscopic hematuria. Immunological assays revealed elevated MPO-ANCA titers (49.2 EU) and positivity for anti-SS-A antibody (37,900 U/mL), in the absence of hypocomplementemia, anti-double-stranded DNA (anti-dsDNA) antibody, anti-SS-B antibody, PR3-ANCA, or anti-glomerular basement membrane (GBM) antibodies. Anti-mitochondrial antibody and hepatitis virus markers, including hepatitis B virus (HBV)-DNA and hepatitis C virus (HCV-RNA), were all negative. Notably, her transaminases, biliary enzymes, and total bilirubin (T-bil) levels were within normal limits. To evaluate potential exocrine gland involvement, ophthalmological examinations were performed, which revealed positive results for both the Schirmer's test and the fluorescein staining test. Based on these objective ocular findings combined with anti-SS-A antibody positivity, the diagnostic criteria for Sjögren’s syndrome (SjS) were fully satisfied. Her renal biopsy findings demonstrated a glomerulus with crescentic formation and global/segmental sclerotic changes, accompanied by diffuse mononuclear cell infiltration in the interstitium (Figure 1a). Crescentic glomerulonephritis characterized by a prominent cellular to fibrocellular crescent was detected (Figure 1b). Based on these histopathological and clinical findings, we ultimately diagnosed the patient with a co-existence of crescentic glomerulonephritis secondary to MPA and tubulointerstitial nephritis induced by SjS. Following her first admission, induction therapy was initiated on hospital day 7 with intravenous methylprednisolone pulse therapy (1,000 mg/day for three consecutive days), followed by oral prednisolone (PSL) at a dose of 50 mg/day. To control the activity of MPA, Rit (500 mg) was administered on hospital days 18 and 25. Additionally, to facilitate a rapid steroid taper while maintaining disease control, concomitant avacopan at a dose of 60 mg/day was introduced on hospital day 26. Moreover, owing to her history of cholelithiasis, concomitant ursodeoxycholic acid (UDCA) at a dose of 150 mg/day was initiated alongside avacopan. Subsequently, a rapid and successful reduction of the PSL dose was achieved within a short period, allowing for her timely discharge. Her renal function significantly improved, with her sCr level decreasing to 1.76 mg/dL. Concurrently, her hematuria and proteinuria were substantially ameliorated, and her MPO-ANCA titers drastically decreased to 4.7 EU (Figure 2).

**Figure 3:**
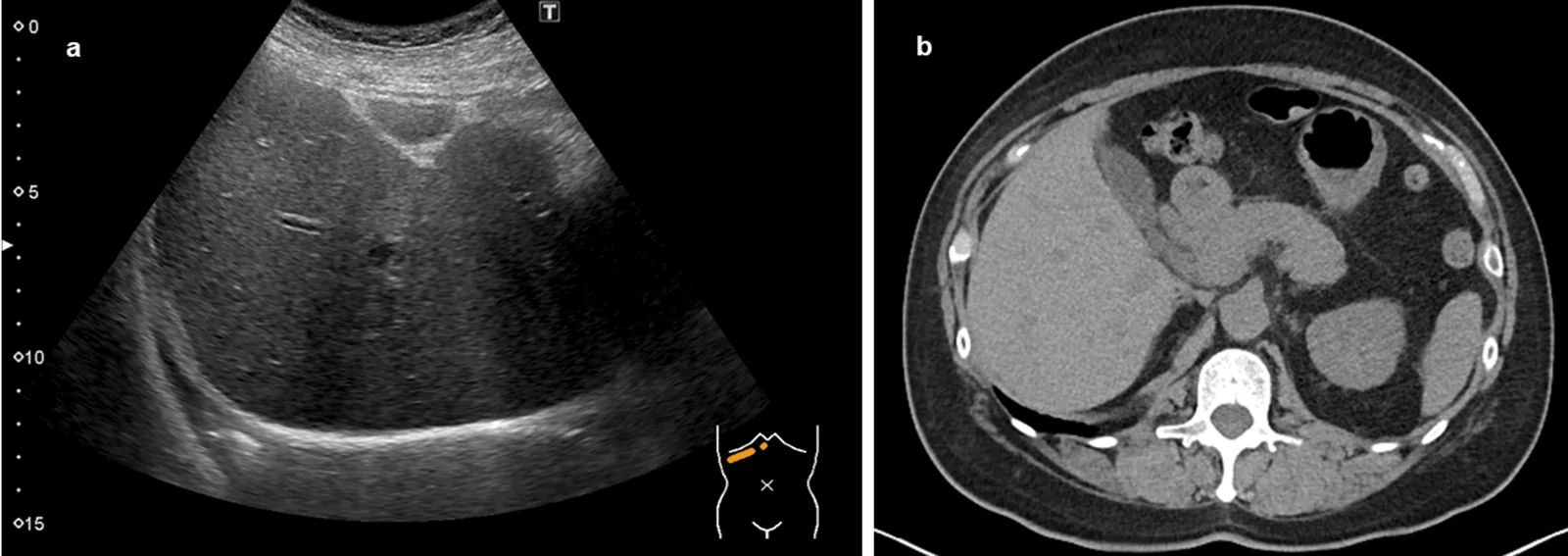
Abdominal images on the second admission. (a) Abdominal ultrasonography shows a smooth liver surface and patent perihepatic spaces without abnormal fluid collection. (b) Contrast-enhanced computed tomography shows no detectable intrahepatic or extrahepatic biliary tract obstruction, structural stricture, or space-occupying lesions.

After her discharge, the patient was closely monitored in our hospital. Over the course of two visits, her clinical course remained uneventful: her renal function was stable without any signs of exacerbation, her MPO-ANCA titers successfully seroconverted to negative, and oral PSL was smoothly tapered to 20 mg/day. However, approximately seven weeks after the initiation of avacopan, she developed a sudden and severe acute liver injury, necessitating emergency readmission. Upon this second admission, her body temperature was 36.1°C, heart rate was 80 beats/minute, and blood pressure was 120/60 mmHg. Her height, body weight, and body mass index were 158 cm, 73 kg, and 29.2 kg/m^2^, respectively. Physical examination revealed no abnormalities in the chest or abdomen, and no peripheral edema was present. There was no cervical lymphadenopathy or joint swelling. The principal laboratory findings upon readmission are presented in Table 1. Laboratory tests revealed marked elevations in transaminases and biliary enzymes: aspartate aminotransferase (AST) was 313 U/L, alanine aminotransferase (ALT) was 488 U/L, and γ-glutamyl transpeptidase (γ-GTP) was 364 U/L, while her direct bilirubin (D-bil) level was initially within the normal range at 0.5 mg/dL. Abdominal ultrasonography revealed a non-dilated extrahepatic bile duct, with no evidence of residual gallstones, biliary sludge, or intraluminal mass lesions. The liver surface appeared smooth, and the adjacent anatomical compartments demonstrated no remarkable abnormalities or ascites (Figure 3a). Additionally, abdominal contrast-enhanced computed tomography was also performed, which ruled out biliary tract obstruction, structural strictures, or space-occupying malignancies in both the intrahepatic and extrahepatic biliary trees (Figure 3b). However, from the second hospital day, her D-bil level began to rise sharply. Other serum biochemistry data showed no remarkable changes except for a significant elevation of total bile acids. Immunological studies revealed no elevation of C-reactive protein, hypocomplementemia, or hypergammaglobulinemia. Negative results were obtained for anti-nuclear antibody, anti-dsDNA antibody, anti-mitochondrial M2 antibody, MPO-ANCA, and PR3-ANCA. Also, serum hepatitis B surface antigen/DNA, anti-hepatitis C virus RNA, and anti-hepatitis A virus IgM antibodies were all negative (Table 1).

**Table 1:** Laboratory findings on the second admission. WBC, white blood cell; Hb, hemoglobin; Plt, platelet; TP, total protein; Alb, albumin; T-bil, total bilirubin; TBA, total bile acid; GOT, aspartate aminotransferase; GPT, alanine aminotransferase; LDH, lactate dehydrogenase; ALP, alkaline phosphatase; γ-GTP, γ-glutamyltransferase; CK, creatine kinase; Amy, amylase; BS, blood sugar; HbA1c, hemoglobin A1c; TG, triglyceride: LDL, low-density lipoprotein cholesterol; HDL, high-density lipoprotein cholesterol; BUN, blood urea nitrogen; Cr, creatinine; UA, uric acid; eGFR, estimated glomerular filtration rate; Na, sodium; K, potassium; Cl, chloride; Ca, calcium; P, phosphorus; CRP, C-reactive protein; IgG, immunoglobulin G; IgA, immunoglobulin A; IgM, immunoglobulin M; C3, component 3; C4, component 4; CH50, total hemolytic complement activity; ANA, antinuclear antibody; anti-ss-DNA Ab; anti-single standard-DNA antibody; anti-ds-DNA Ab; anti-double standard-DNA antibody; MPO-ANCA, myeloperoxidase antineutrophil cytoplasmic antibody; PR3-ANCA, proteinase 3 antineutrophil cytoplasmic antibody; anti-CL Ab, anti-cardiolipin IgG antibody; EB anti-VCA IgG FA, Epstein-Barr virus anti-viral capsid antigen IgG fluorescent antibody; EB anti-VCA IgA FA, Epstein-Barr virus anti-viral capsid antigen IgA fluorescent antibody; EB anti-EBNA FA, Epstein-Barr virus anti-Epstein-Barr virus nuclear antigen 1 fluorescent antibody; IgM-HA Ab, anti-hepatitis A virus IgM antibody; IgA-HA Ab, anti-hepatitis A virus IgA antibody; CMV, cytomegalovirus; EIA, enzyme immunoassay; COI, cutoff index

Parameter	Unit	Value	Reference range
Hematology			
WBC count	µL^-1^	7,000	3,300-8,600
Hemoglobin	g/dL	12.7	13.7-16.8
Platelet count	10^4^/µL	25.3	15.8-34.8
Blood chemistry			
TP	g/dL	6.3	6.0-8.0
Alb	g/dL	3.4	4.1-5.1
T-bil	mg/dL	0.8	0.2-1.2
D-bil	mg/dL	0.5	0.2-1.2
GOT	IU/L	313	10-40
GPT	IU/L	488	10-40
TBA	μmol/L	273.4	<10
LDH	IU/L	350	140-280
ALP	IU/L	146	38-113
γ-GTP	IU/L	364	9-32
CK	IU/L	33	41-153
Amy	IU/L	96	44-132
BS	mg/dL	96	73-109
HbA1c	%	5.5	4.6-6.2
TG	mg/dL	110	30-149
LDL	mg/dL	148	65-139
HDL	mg/dL	140	40-103
BUN	mg/dL	31.7	8.0-20.0
Cr	mg/dL	1.77	0.6-1.0
eGFR	mL/min	24	>60
UA	Mg/dL	5.6	2.6-7.0
Na	mEq/L	141	138-145
K	mEq/L	4.3	3.5-5.0
Cl	mEq/L	109.0	101-108
Ca	mg/dL	9.0	8.8-10.0
P	mg/dL	3.1	2.5-4.7
Immunological test			
CRP	mg/dL	0.13	0.0-0.3
Ferritin	ng/dL	293	15-150
IgG	mg/dL	1,192	870-1,700
IgA	mg/dL	477	110-410
IgM	mg/dL	90	52-270
C3	mg/dL	81	80-140
C4	mg/dL	19	15-45
CH50	U/mL	54	30-45
ANA	titer	x80	<40
Anti-ss-DNA Ab	IU/mL	Negative	<10
Anti-ds-DNA Ab	IU/mL	Negative	<10
Anti-SS-A/Ro antibody	U/mL	11,000	<10
Anti-SS-B/La antibody	U/mL	Negative	<10
MPO-ANCA	IU/mL	Negative	<1
PR3-ANCA	IU/mL	Negative	<1
Anti-CL Ab	IU/mL	Negative	<12.3
Anti-mitochondrial M2 Ab	U/mL	Negative	<5.0
Hepatitis virus test			
HBV-DNA	logIU/mL	Negative	
HCV-RNA	logIU/mL	Negative	
EB anti-VCA IgG FA	EIA	_X_80	
EB anti-VCA IgA FA	EIA	Negative	
EB anti-EBNA FA	EIA	Negative	
IgM-HA Ab	COI	Negative	
IgA-HA Ab	COI	Negative	
CMV antigenemia	cells/slide	Negative	

**Figure 4:**
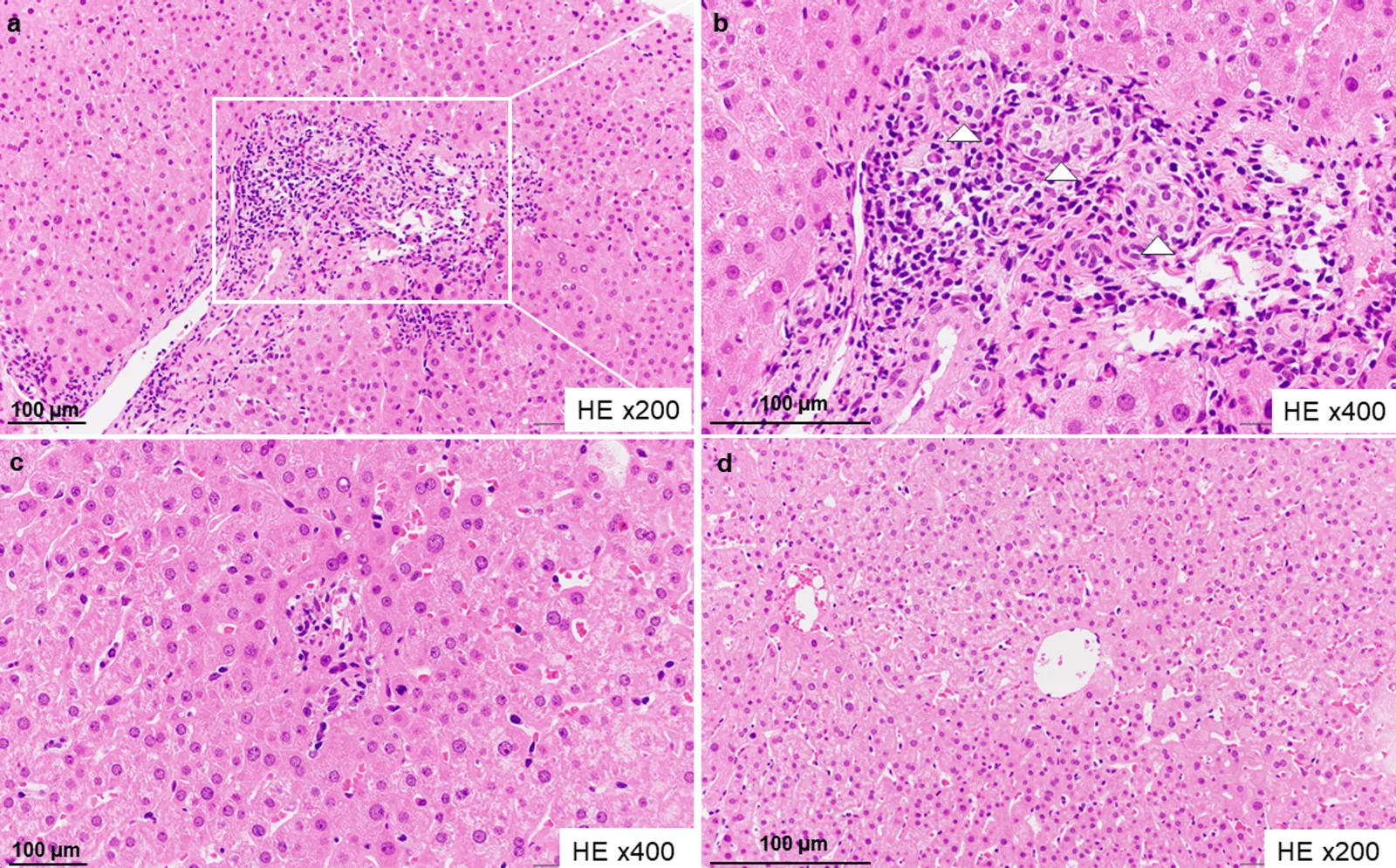
Histopathological findings of liver biopsy specimens based on hematoxylin and eosin (HE) staining on hospital day 3. (a) Low-power magnification shows significant inflammatory cell infiltration within and around the portal tract (Glisson’s sheath) (HE, ×200). (b) High-power view of the boxed area in (a) demonstrates severe round cell infiltration with a notable absence of eosinophils. Destruction of the bile duct and inflammatory infiltration into the biliary epithelium are observed, characteristic of a florid duct lesion (arrowheads) (HE, ×400). (c) High-power view of the liver parenchyma shows a focus of spotty necrosis in some hepatocytes (HE, ×400). (d) Low-power view of the pericentral area (around the central vein) shows no apparent inflammation or parenchymal destruction (HE, ×200).

**Figure 5:**
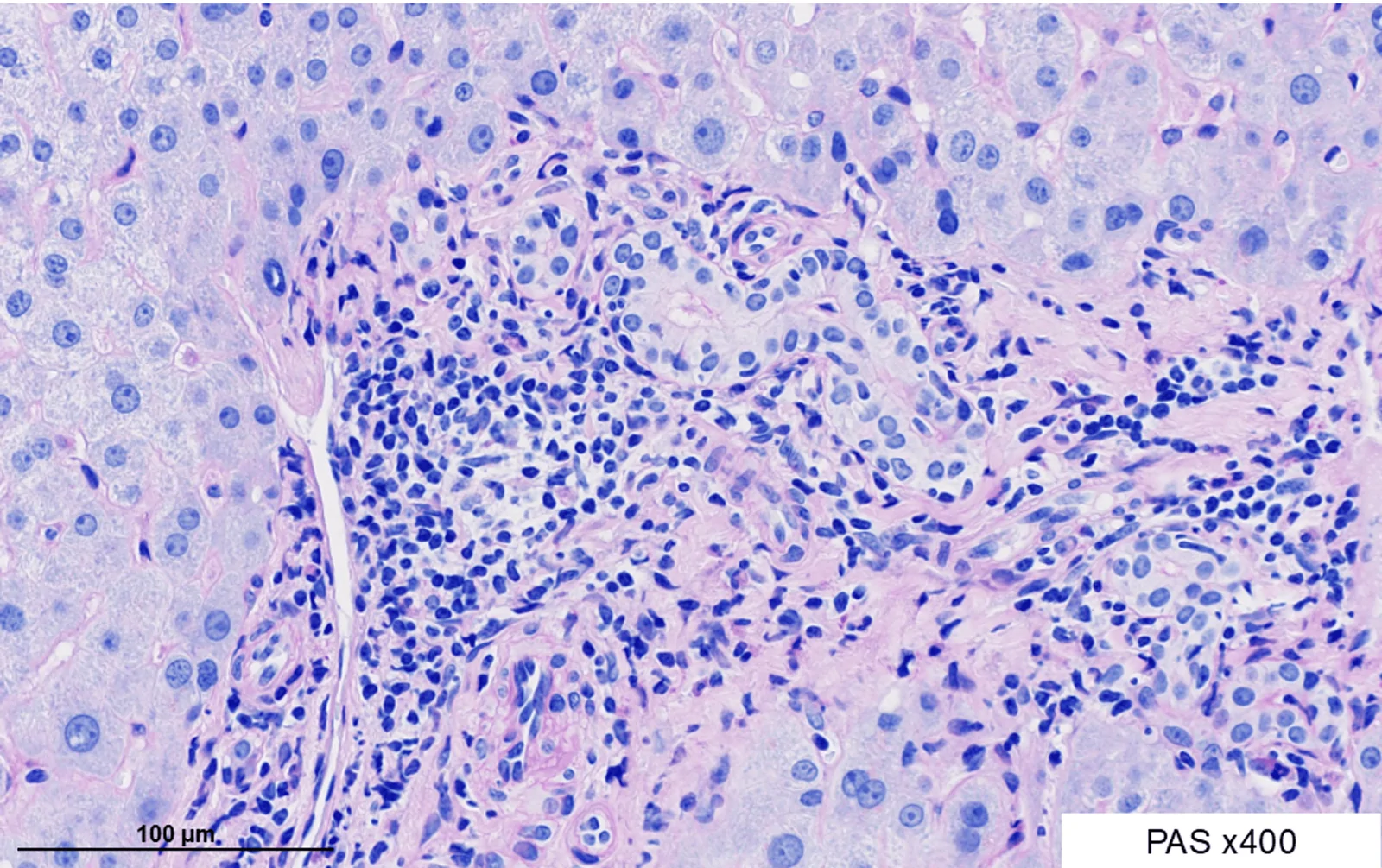
Histopathological findings of the portal tract stained with periodic acid-Schiff (PAS). High-power view demonstrating severe inflammatory cell infiltration into the portal tract (Glisson's sheath) without significant enlargement or expansion of the portal area. Consistent with the H&E findings, marked destruction of the bile duct epithelium and prominent inflammatory cell infiltration into the biliary architecture are clearly observed (PAS, ×400).

**Figure 6:**
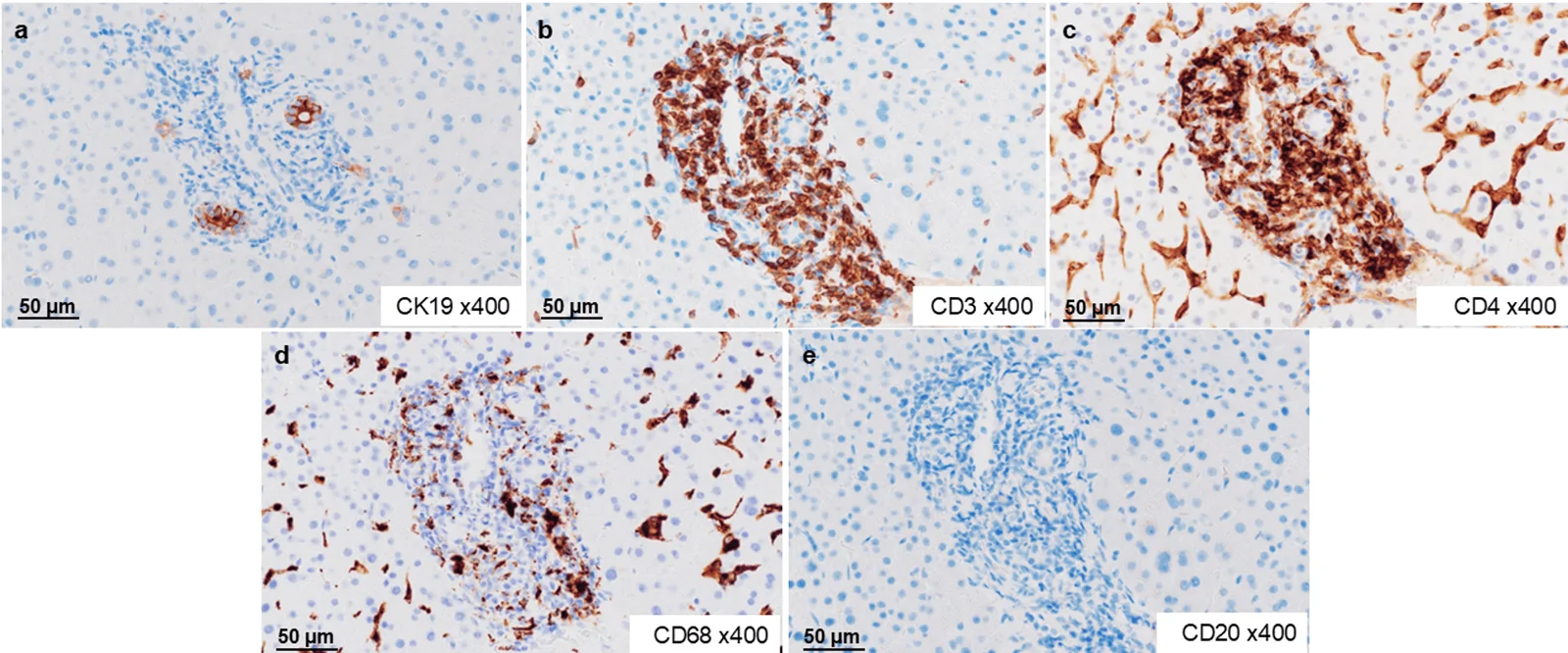
Immunohistochemical analysis of inflammatory infiltrates in damaged liver tissue. (a) Cytokeratin 19 (CK19) staining, a marker for biliary epithelial cells, reveals attenuation and patchy loss of CK19 immunoreactivity (original magnification ×400). (b, c) Intense infiltration of CD3-positive (b) and CD4-positive (c) T lymphocytes is observed at the injured site, predominantly clustering around the actively damaged bile ducts (original magnification ×400). (d) CD68-positive macrophages show moderate infiltration within the portal tract (original magnification ×400). (e) CD20-positive B cells are notably absent, with no significant infiltration (original magnification ×400).

To elucidate the etiology of this rapid hepatic deterioration, a percutaneous liver biopsy was performed on hospital day 3 (Figures 4a-4d, 5). Histopathological evaluation demonstrated marked inflammatory cell infiltration within and around the portal tract (Glisson’s sheath) (Figure 4a). High-power views revealed significant round cell infiltration with a notable absence of eosinophils, along with bile duct destruction and inflammatory infiltration into the biliary epithelium, characteristic of a florid duct lesion (Figures 4b, 5). In the liver parenchyma, foci of spotty necrosis were observed in some hepatocytes (Figure 4c). In contrast, no apparent inflammation or parenchymal destruction was detected in the pericentral area around the central vein (Figure 4d). Immunohistochemical staining for cytokeratin 19 (CK19) highlighted the damaged interlobular bile ducts, demonstrating attenuated cytoplasmic staining intensity in a subset of biliary epithelial cells, along with an irregular arrangement and partial loss of the epithelium (Figure 6a). Furthermore, immunohistochemical analysis of the inflammatory infiltrates revealed that significant number of CD3- or CD4-positive T cells (Figures 6b, 6c), as well as moderate number of CD68-positive macrophages (Figure 6d), had accumulated within the damaged and inflamed liver tissues. CD20-positive cells were not detected around the damaged liver tissues (Figure 6e). Based on these biopsy findings, avacopan was immediately discontinued. Given the high degree of inflammatory cell infiltration observed in the liver biopsy specimen, the patient was placed on strict fasting (nil per os), and the dose of oral PSL was temporarily increased to 30 mg/day. Furthermore, intensive medical management was instituted: the dosage of UDCA was optimized to its maximum capacity of 600 mg/day, and daily intravenous injections of Stronger Neo-Minophagen C were commenced. Under this aggressive regimen, her T-bil level peaked transiently at 4.0 mg/dL on hospital day 7 but subsequently demonstrated a progressive decline, decreasing to 1.2 mg/dL by hospital day 18, which allowed for her safe discharge. By hospital day 35, her T-bil, AST, and ALT levels had all completely returned to their normal reference ranges, accompanied by sustained stabilization of her renal function (Figure 2).

## Discussion

In the present case, we provide the clinicopathological findings of NSDC caused by avacopan therapy for MPA. To our knowledge, this is the first report to demonstrate detailed immunohistochemical analysis of a liver biopsy specimen, offering crucial insights into its inflammatory profile and the underlying pathogenesis of destructive cholangitis caused by avacopan. Furthermore, we comprehensively reviewed seven previously reported cases of biopsy-proven avacopan-induced cholangitis to elucidate the clinical features of this severe DILI (Table 2). Critically, the pathological features of the present case did not meet the criteria for VBDS, which has been widely suggested in the previous literature; instead, our case represented NSDC, which likely reflects a precursor state to VBDS. Crucially, a meticulous review of prior cases categorized as VBDS revealed that, while they undoubtedly exhibited severe destructive cholangitis, true "vanishing" or profound loss of bile ducts, which carries an extremely poor prognosis, was not definitively proven. Therefore, we propose that NSDC is a more precise and accurate pathological diagnosis that faithfully reflects the true nature of this drug-induced biliary lesion.

**Table 2:** Clinical features of previously reported cases of biopsy-proven cholangitis caused by avacopan treatment in patients with ANCA-associated vasculitis. ANCA, antineutrophil cytoplasmic antibody; AAV, ANCA-associated vasculitis; ALT, alanine aminotransferase; AST, aspartate aminotransferase; BMI, body mass index; DILI, drug-induced liver injury; F, female; iv, intravenous; γ-GTP, gamma-glutamyl transpeptidase; GPA, granulomatosis with polyangiitis; M, men; MPA, microscopic polyangiitis; MPO, myeloperoxidase; NA, not available; PSL, prednisolone; Ref, reference; sCr, serum creatinine; SNMC, stronger neo-minophagen C; T-bil, total bilirubin; UDCA, ursodeoxycholic acid; yr, year.

Case Ref No.	AAV type/ANCA subtypes	Age (yr)/Sex	BMI (kg/m^2^)	Pre-treatment with Rit	ANCA at onset (EU)	Peak GOT/GPT/γGTP/T-bil at onset (U/L/U/L/U/L/mg/dL)	Time to DILI onset/recovery (days)	Discontinuation of avacopan	Treatment	Outcome
Uchida et al. (2024) [14]	MPA MPO-ANCA	NA	NA	Yes	NA	395/624/507/14.1	31/257	Yes	NA	Recover
Mori et al. (2025) [15]	MPA MPO-ANCA	75/F	15.4	Yes	NA	155/357/352/5.3	14/	Yes	UDCA (unknown dose), orally, Inchinkoto	Death
Yamaguchi et al. (2024) [17]	MPA MPO-ANCA	75/F	NA	Yes	NA	218/565/654/7.7	45/150	Yes	Dose up PSL to 40 mg/day, UDCA 900 mg/day	Recover
Shirota et al. (2024) [19]	MPA MPO-ANCA	70/F	NA	Yes	NA	155/357/352/3.6	40//90	Yes	PSL 7.5 mg was continued; UDCA 900 mg/day,	Recover
Kojima et al. (2024) [21]	GPA MPO-ANCA	76/F	NA	Yes	Negative	395/624/NA/14.1	28/156	Yes	Cessation of PSL, UDCA 600 mg/day	Recover
Yamaguchi et al. (2025) [22]	MPA MPO-ANCA	84/F	NA	Yes	Negative	273/490/442/7.9	60/93	Yes	Cessation of PSL	Persistent DILI
Hishinuma et al. (2026) [23]	MPA MPO-ANCA	81/M	20.3	Yes	NA	220/274//596/3.1	42/	Yes	mPSL pulse 1,000 mg x 3 days followed by PSL 60 mg/day, and UDCA 600 mg/day	Death
This case	MPA MPO-ANCA	55/F	29.2	Yes	Negative	313/488/747/4.8	48/14	Yes	Dose up PSL to 30 mg/day, UDCA 600 mg/day, and daily IV of SNMC	Recover

Regarding the diagnostic plausibility of avacopan-induced cholangitis in our patient, several key factors strongly support this conclusion: (1) the relatively early onset of severe DILI after initiation of avacopan treatment; (2) prompt amelioration of DILI following withdrawal of avacopan combined with intensified supportive treatment; and (3) exclusion of other potential etiologies of destructive cholangitis, such as primary biliary cholangitis (PBC), viral hepatitis, alcohol abuse, or alternative concomitant medications. In this case, a low-dose sulfamethoxazole/trimethoprim (SMX/TMP) regimen had been prescribed for six weeks as prophylaxis against Pneumocystis pneumonia during the intensive immunosuppressive induction phase. However, upon tapering the oral PSL dose to 20 mg/day or less, SMX/TMP was discontinued approximately two weeks prior to the onset of liver injury, effectively ruling it out as a causative agent. Furthermore, although the patient fully satisfied the diagnostic criteria for SjS, an SjS-related hepatic manifestation was highly unlikely. Her systemic inflammatory responses and SjS clinical activity were completely suppressed by the ongoing intensive immunosuppressive therapy, and the hepatic deterioration occurred in the absence of dry eyes or dry mouth. Consequently, avacopan remains the most likely and primary culprit behind this severe biliary injury.

As described above, the randomized controlled trial demonstrated that avacopan was generally well tolerated, with few serious hepatic adverse events [7]. In contrast, recent real-world data have revealed a markedly higher incidence of avacopan-associated liver injury in Japanese cohorts than in Western populations. Specifically, recent reports indicate that the incidence of hepatotoxicity was 0% in Spain [25], 4.3% in the United States [13], 5.1% in Germany [26], and 16.7% to 38.1% in Japan [14-16], underscoring a notably higher frequency among Japanese patients. These stark discrepancies strongly suggest that population-specific factors, including ethnic or genetic predispositions and advanced age, may underlie this increased risk in Japan. However, while clinical reports of avacopan-induced liver injury are increasing, cases definitively diagnosed as severe cholangitis via histopathological evaluation remain exceedingly rare. To date, including our patient, only eight cases of biopsy-proven avacopan-induced cholangitis have been documented worldwide (Table 2). This point underscores that serological liver function tests alone are insufficient to distinguish transient hepatotoxicity from progressive biliary destruction. Therefore, prompt liver biopsy is indispensable, not only to establish an accurate diagnosis of VBDS but also to guide timely, aggressive therapeutic interventions to prevent irreversible biliary and chronic liver injury.

Thus far, several clinical baseline characteristics have been proposed as critical risk factors for avacopan-induced liver injury. Recent Japanese cohort studies suggest that advanced age, low BMI, persistent MPO-ANCA positivity reflecting poorly controlled MPA, and early onset of liver dysfunction (typically within the first month of treatment) are significantly associated with severe DILI and biliary phenotypes [15,16]. However, as shown in Table 2, a meticulous review of the eight biopsy-proven cholangitis cases documented to date, including our patient, reveals that these proposed risk factors are surprisingly inconsistent with the actual clinical presentation or outcomes. Most notably, our patient completely deviated from this high-risk profile, as she was middle-aged, obese, and had achieved full clinical and serological remission of MPO-ANCA by the time of the later onset at seven weeks. This critical discrepancy demonstrates that severe avacopan-induced cholangitis could occur in any clinical setting, regardless of baseline patient phenotype or disease activity. Furthermore, the clinical outcomes of these biopsy-proven cases highlight the potentially dismal prognosis of this condition; among the eight reported patients, two died and one suffered from persistent long-term liver injury. Regarding therapeutic interventions, intensification of UDCA and temporary dose escalation of PSL, as successfully implemented in our patient, appear to be beneficial in several surviving cases (Table 2). However, due to the extremely limited number of cases, a definitive treatment consensus cannot be established, and the true efficacy of these strategies remains to be validated. Collectively, rather than being driven merely by patient-specific vulnerabilities, the pathogenesis of this severe DILI is highly likely to derive from the intrinsic pharmacological effects or mechanisms of avacopan within the context of AAV.

Recent literature suggests that avacopan-induced DILI is more prevalent during the remission induction phase, particularly when avacopan is combined with Rit [15,23,24]. Crucially, as summarized in Table 2, all documented patients with biopsy-proven severe cholangitis due to avacopan therapy, including our patient, had received prior Rit therapy. This clinical association aligns with observations by Hishida and Nagata, who noted that severe hepatotoxicity, marked by profound hyperbilirubinemia or overt VBDS, predominantly occurred in patients treated with Rit [24]. Our detailed immunohistochemical analysis of the liver biopsy provides a pivotal clue to this pathogenesis. The profound infiltration of CD4-positive T cells and moderate infiltration of CD68-positive macrophages, contrasted with the complete absence of CD20-positive B cells and eosinophils, strongly implies that the underlying mechanism is not driven by B cell-mediated autoimmunity or classical allergic drug hypersensitivity. Instead, it likely involves a drug-related, aberrant cellular immune reaction. Clinically, the timeline of these events is highly telling, as the majority of the cases reported in Table 2 developed during the tapering of oral PSL. We speculate that the potent immunosuppression achieved by preceding Rit therapy allows for a rapid and aggressive tapering of PSL. However, this accelerated withdrawal of steroids may inadvertently unmask and accelerate an avacopan-induced, T-cell-mediated aberrant immune response. This hypothesis is supported by the fact that prompt treatment with increased doses of PSL, as successfully implemented in our case, reversed the process and yielded favorable outcomes. From a pathological perspective, despite the absence of autoantibodies and CD20-positive B cells, the NSDC observed in our case apparently resembles the developmental stages of PBC. Specifically, our findings seem to capture a transitional phase, spanning from early PBC lesions, characterized by florid duct lesions and ductular proliferative changes, to irreversible terminal VBDS featuring bridging necrosis and septal fibrosis [27]. Consequently, further investigations are warranted to clarify the synergistic relationship between preceding Rit therapy and avacopan exposure, as well as to fully elucidate the exact molecular mechanisms driving this severe biliary destruction.

reover, recent articles indicate that genetic backgrounds significantly influence susceptibility to avacopan-induced liver injury [15,23,24]. Notably, the CYP3A53 allele is highly prevalent in the Japanese population [28]. Because this allele causes a complete loss of CYP3A5 activity, the majority of Japanese individuals lack functional CYP3A5 and rely almost entirely on CYP3A4 for hepatic drug metabolism [24,28]. Under these genetic constraints, certain CYP3A4 polymorphisms can further diminish baseline enzyme function [29]. Because avacopan is primarily metabolized by CYP3A4 [30], this may potentially increase vulnerability to its toxicities. Thus, co-administration with CYP3A4 inhibitors (e.g., vonoprazan) or inducers (such as rifampin) markedly alters avacopan exposure, while avacopan itself increases the plasma levels of other CYP3A4 substrates [30]. Taken together, because most Japanese patients lack functional CYP3A5, their avacopan metabolism may be uniquely susceptible to changes in CYP3A4 activity driven by drug-drug interactions (DDIs) or genetic variants [15,28,29]. Careful pre-treatment DDI assessment and strict clinical monitoring are essential in populations with high CYP3A53 homozygosity.

Although several mechanisms may underlie avacopan-associated liver injury, the selective inhibition of the C5aR1 by avacopan is reported to induce biliary and hepatic regeneration. Animal studies have demonstrated that C5aR1-deficient mice exhibit aggravated liver injury, reduced compensatory hepatocyte proliferation, and increased apoptosis following toxic insults [31,32]. This impaired regenerative capacity may explain the persistent, progressive ductopenia and prolonged cholestasis observed in severe cases of avacopan-induced liver injury, including our present case. More importantly, these findings offer an intriguing pathophysiological insight into the unique affinity of the biliary system to avacopan. We hypothesize that the targeted blockade of C5aR1 by avacopan disrupts the intrinsic remodeling and homeostatic mechanisms naturally maintained by cholangiocytes. This specific mechanism may elucidate why severe cholangitis has been distinctly linked to avacopan, whereas no such cases have been reported with other complement inhibitors, such as iptacopan (a factor B inhibitor) or ravulizumab (an anti-C5 monoclonal antibody).

Reflecting these serious safety concerns regarding avacopan, emerging updated guidelines worldwide, including those from the U.S. Food and Drug Administration (FDA), European consortia, and Japanese consensus statements, increasingly recommend caution or avoidance of avacopan during the initial remission induction phase for AAV. Furthermore, these hepatotoxicity concerns have had a substantial academic and social impact, culminating in the recent retraction of the representative ADVOCATE trial [8] due to data integrity issues surrounding these hepatic adverse events.

## Conclusions

Our patient's deviation from previously proposed risk profiles indicates that avacopan-induced severe hepatotoxicity and cholangitis can occur in any clinical setting, regardless of baseline phenotypes. We hypothesize that, in patients with an inherent genetic predisposition, such as population-specific variations in drug-metabolizing enzymes or C5aR1-related signaling pathways within the biliary system, administration of avacopan triggers a profound disruption of T-cell-mediated cellular immunity, provoking severe cholangitis that ultimately transitions into VBDS. Given these critical developments and the high potential for irreversible biliary damage, the initiation or concomitant use of avacopan must be approached with extreme caution, and rigorous, accurate post-marketing surveillance remains imperative.
